# A Constrained Layer Damping Perspective on Floating Floor Systems for Low-Frequency Impact Noise Control

**DOI:** 10.3390/polym18131606

**Published:** 2026-06-28

**Authors:** Yinghui Jiao, Junhuai Xu, Yaohan Feng, Haoshuai Suo, Yangang Zhang, Yanli Nan, Xiao Wang, Dongsheng Liu, Ya Feng, Pengfei Si

**Affiliations:** 1 College of Geography and Planning, Chengdu University of Technology, Chengdu 610059, China; jiaoyinghui08@cdut.edu.cn; 2 China Southwest Architecture Design and Research Institute Co. Ltd., CSCEC Energy Conservation Materials and Equipment Engineering Research Center, Chengdu 610041, China; xujunhuaiup@163.com (J.X.); 18223193710@163.com (Y.F.); shs95212022@163.com (H.S.); zhangyangang0817@163.com (Y.Z.); nan-1027@163.com (Y.N.); wangxlh666@163.com (X.W.); boydirk2010@163.com (D.L.); xnyjd2@vip.163.com (Y.F.); 3 CSWADI (Sichuan) Science and Technology Co., Ltd., Chengdu 610041, China

**Keywords:** floating floor systems, low-frequency impact noise, constrained-layer-damping-inspired, composite underlayments, damping sound insulation material

## Abstract

Low-frequency impact sound control remains a critical challenge for floating floor systems. Conventional resilient underlayment materials exhibit insufficient damping and are prone to long-term deformation, making stable low-frequency sound insulation difficult to achieve. This study presents the development of a composite floating floor underlayment comprising recycled rubber granules, polymer resin, and quartz sand. Based on the constrained layer damping-inspired (CLD-inspired) perspective, the vibration attenuation and noise reduction mechanism is elucidated, and the material’s physical properties, mechanical behavior, microstructure, and acoustic performance are systematically investigated. The results indicate that excessively large rubber granules aggravate curing shrinkage cracking. Optimal processing characteristics are achieved with a binder content of 20 wt% and a rubber granule size of 50 mesh. Laboratory characterization reveals that, compared with conventional cross-linked polyethylene (XLPE) foam underlayments, the proposed composite underlayment reduces the impact sound pressure level by an average of 3–5 dB in the low-frequency band below 250 Hz, and the overall sound insulation performance is improved by 10.77%. Dynamic mechanical analysis shows the composite storage modulus declines from 280 MPa at −20 °C to 10 MPa at 80 °C, while the loss factor remains above 0.2 under typical indoor conditions. Such stable viscoelastic behavior enables efficient shear dissipation of low-frequency vibration energy under the CLD-inspired mechanism. Full-scale field testing combined with long-term observation over 3000 loading cycles demonstrates excellent structural compatibility between the underlayment and the gypsum screed, with no cracking or appreciable deformation observed during prolonged service. The weighted impact sound improvement index (ΔLw) attains 15 dB. These findings verify that the CLD-inspired composite underlayment simultaneously achieves efficient low-frequency impact sound control and superior long-term structural stability, providing an innovative material solution and design strategy for impact noise mitigation in residential floating floor applications.

## 1. Introduction

Impact noise generated by footsteps, furniture movement, and children running presents a persistent challenge in residential buildings [1]. Unlike airborne noise, impact noise is predominantly structure-borne and concentrated in the low-frequency range below 250 Hz [2,3], where vibrational energy propagates efficiently through structural elements and is inherently difficult to attenuate [4,5,6]. Prolonged exposure to such noise has been identified by the World Health Organization (WHO) as an environmental stressor associated with annoyance, sleep disturbance, and adverse health outcomes [7,8,9].

With increasing urban density and the widespread adoption of multi-storey residential construction, complaints related to floor impact noise remain among the most frequent causes of post-occupancy dissatisfaction and the need for retrofit interventions [10,11]. Floating floor systems are therefore widely adopted to mitigate impact noise transmission by introducing a resilient layer between the structural slab and the finished surface [12,13,14]. In practice, however, their effectiveness is often limited, particularly in the low-frequency range that dominates residential impact noise [14,15,16].

Commonly used resilient underlayments, such as cross-linked polyethylene (XLPE) foams and polyurethane foam mats, rely primarily on compliance and porosity to insulate impact noise. While these materials can provide attenuation at mid- to high-frequency ranges, their low density and limited internal damping restrict their effectiveness at low frequencies [17,18,19,20]. In addition, highly compliant underlayments are susceptible to long-term creep and permanent deformation under sustained service loads. Such deformation may induce stress concentrations within brittle finishing layers, including gypsum screeds or ceramic tiles, leading to surface cracking, delamination, and premature failure. To compensate for these deficiencies, designers often resort to increasing slab thickness, adding reinforcement, or reducing floor-to-ceiling height [21,22], thereby increasing construction cost and complexity without addressing the underlying physical limitations of the resilient layer.

These limitations stem largely from the simplified manner in which the mechanical behavior of floating floor systems is commonly interpreted. Floating floors are typically represented using mass-spring models [23,24,25,26], in which improved acoustic performance is primarily attributed to increased compliance of the resilient layer. While such models provide useful first-order insight, they provide limited guidance for balancing acoustic performance against structural serviceability, particularly under low-frequency excitation and long-term loading conditions. For the soft resilient materials, prolonged use will significantly reduce their noise reduction performance [27].

From a mechanical perspective, a floating floor assembly resembles a CLD-inspired layered system [28,29,30,31], consisting of two relatively rigid layers-the structural slab and the finished layer-separated by a viscoelastic interlayer. Under impact loading, the relative motion between rigid layers induces shear deformation within the interlayer, dissipating vibrational energy through viscoelastic losses and interfacial friction [32,33,34]. Although this configuration departs from the classical CLD conventionally defined in mechanical engineering, it shares the essential functional characteristic of shear-dominated energy dissipation within a layered structure [35,36,37,38].

When viewed through a CLD-inspired perspective [39], the design requirements of the resilient interlayer differ from those associated with conventional soft isolation strategies. Rather than solely minimizing stiffness, the interlayer must provide a balanced combination of mass density, internal damping capacity, and elastic modulus [40,41]. Mass contributes to low-frequency sound insulation, damping governs energy dissipation under dynamic loading, and elastic modulus controls load-bearing capacity and mechanical compatibility with rigid finishing layers [42]. Failure to balance these parameters risks degrading either acoustic performance or structural reliability. It should be noted that recycled rubber has been employed in acoustic materials, most notably in porous absorbers fabricated from shredded waste tires [43], where sound energy is dissipated through viscous and thermal losses within interconnected pores. By contrast, the present composite underlayment is designed as a dense, thin-layer system in which energy dissipation relies on constrained viscoelastic deformation and interfacial shear—a fundamentally different mechanism targeting structure-borne impact noise insulation rather than airborne sound absorption.

Composite underlayments offer a means to independently tailor mass, stiffness, and damping—parameters that are inherently coupled in homogeneous materials. Heterogeneous composites incorporating viscoelastic matrices and rigid fillers are particularly attractive, as they allow the mechanical response to be tuned while preserving sufficient damping capacity [44,45,46,47]. Such approaches also address serviceability issues, including long-term creep deformation and surface cracking, which remain persistent challenges in floating floor systems.

The objective of this study is to investigate a CLD-inspired design framework for floating floor underlayment design and to evaluate its implications for low-frequency impact noise control and structural compatibility in residential buildings. A thin composite underlayment based on recycled tire rubber and mineral fillers is developed and assessed through material characterization, laboratory-scale impact sound testing, and full-scale field measurements. By interpreting the floating floor from a CLD-inspired perspective, this work provides physical insight into the coupled acoustic–mechanical behavior of the assembly and derives transferable design guidelines for resilient underlayments in building applications.

## 2. Materials and Methods

### 2.1. Raw Materials

End-of-life tire rubber (China Rubber Regeneration (Suzhou) Co., Ltd., Suzhou, China) was mechanically processed into crumb particles with nominal sizes ranging from 10 to 200 mesh (approximately 0.08–2 mm) and served as the primary viscoelastic phase. Quartz sand (Jiangsu Pacific Quartz Co., Ltd. Lianyungang, China) with a median particle size of approximately 200 μm was employed as a rigid filler to adjust mass density and stiffness. A bio-based polyester resin (Wankai New Materials Co., Ltd., Haining, China) was used as the binder to ensure adequate cohesion between phases while maintaining viscoelastic behavior under service-relevant conditions. All materials were used as received without further chemical modification.

### 2.2. Composite Fabrication

Composite underlayments were fabricated by mechanically mixing rubber particles, quartz sand, and polymer binder to obtain a homogeneous mixture. The mixture was subsequently cast into steel molds and cured under ambient conditions. The thickness of all specimens was controlled at 4 mm, corresponding to typical requirements for floating floor applications.

Prior to systematic performance evaluation, a series of preliminary screening experiments was conducted to define a stable fabrication window. These experiments focused on the influence of rubber particle size and binder content on formability and dimensional stability. Binary composite materials (RC) were prepared by mixing rubber particles with resin, followed by molding and curing. For Experimental Group 1, the rubber particle size was fixed at 50 mesh, while the resin content was varied at 5%, 10%, 20%, and 30%. For Experimental Group 2, the resin content was maintained at 20%, and the rubber particle sizes were varied at 10, 20, 50, 100, and 200 mesh. It was observed that excessive binder content resulted in pronounced curing-induced shrinkage, leading to dimensional instability. Conversely, composites prepared with excessively fine rubber particles exhibited an increased tendency toward cracking. This behavior is attributed to non-uniform stress development within the polymer-rich matrix during curing, rather than cracking of any mineral or cementitious phase.

**Figure 1:**
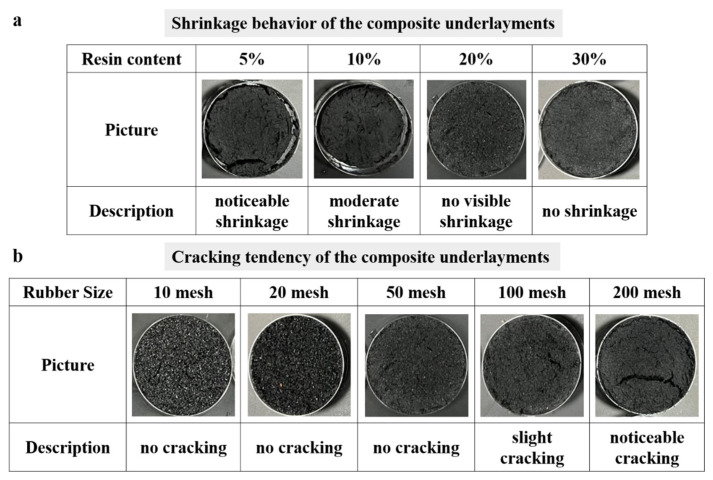
Preliminary screening experiments: (**a**) effect of resin content on shrinkage behavior of the composite underlayments; (**b**) influence of rubber particle size on cracking tendency of composites.

These observations indicate that both binder content and rubber particle size must be carefully controlled to ensure reproducible fabrication and structural integrity of the composite underlayments. Representative examples illustrating the effects of processing parameters on material integrity and cracking behavior are presented in Figure 1.

Based on the processing window established above, a series of ternary composite formulations was prepared for systematic investigation. The ternary composites were obtained by partially substituting rubber particles with quartz sand while maintaining a fixed resin content of 20%. The quartz sand substitution ratios were 0%, 5%, 10%, 15%, 20%, 25%, and 30%, and the corresponding materials were designated as RC, R5-RC, R10-RC, R15-RC, R20-RC, R25-RC, and R30-RC, respectively. All specimens used for subsequent testing were cured under identical conditions (23 °C ± 1 °C; RH 50 ± 5%) and stored for at least 28 days prior to characterization to ensure stable material properties.

### 2.3. Physical Characterization

The theoretical density of the composite materials was calculated using a linear mixing rule [48] based on the mass fractions and densities of the constituent phases. Bulk density was experimentally determined in accordance with GB/T 1033.1-2018 by measuring the mass and volume of representative specimens [49].

Thermal conductivity was measured using a heat flow meter apparatus in accordance with GB/T 10295-2008 to assess compatibility with building envelope requirements. Measurements were conducted at controlled temperatures, and average values were reported [50].

Water retention behavior was evaluated in accordance with ASTM D570-98 using cylindrical specimens with dimensions of 30 mm in length and 25 mm in diameter [51]. Both short-term (24 h immersion) and long-term water absorption tests were conducted. For long-term testing, specimens were periodically removed from water, surface moisture was gently removed, and mass was measured to an accuracy of 0.001 g until saturation equilibrium was reached. The water retention percentage (WR%) was calculated based on mass change relative to the dry state.

To describe the time-dependent water absorption behavior, a three-parameter empirical model [52] was employed to fit the experimental data. Model parameters were obtained using MATLAB R2018b, and the quality of fit was evaluated using the coefficient of determination (R^2^).

**Figure 2:**
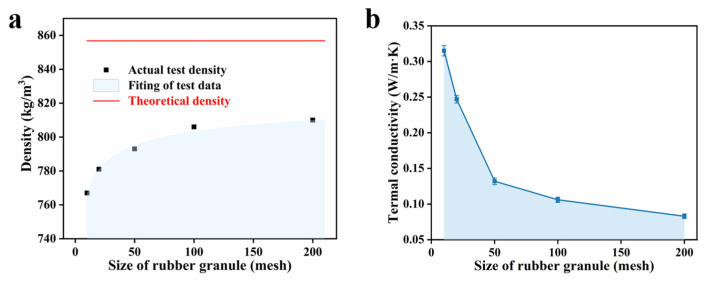
Test data from the preliminary pre-experiment: (**a**) Density changes for different rubber particle sizes. (**b**) Thermal conductivity changes for different rubber particle sizes.

To assist in further screening the optimal material ratio, a series of physical characterizations for the pre-experiment were also carried out simultaneously (Figure 2 and Table 1). For each material ratio, three to five replicate specimens were tested, and the reported values represent the arithmetic mean.

The systematic characterization of density, thermal conductivity, and water absorption aims to verify that the material meets fundamental building specifications and directly serves the core objectives of this study: density and thermal conductivity data relate to both building applicability and the mass parameters in CLD-inspired design, while water absorption behavior reveals the material’s potential durability under humid service conditions. Together, these parameters underpin the design of composite materials that balance acoustic performance with long-term stability.

### 2.4. Mechanical Characterization

Static mechanical properties were evaluated through uniaxial compression and tensile tests using a universal testing machine at a displacement rate of 1 mm/min. The apparent elastic modulus was determined from the initial linear region of the stress–strain curves.

To assess long-term mechanical stability under service-relevant conditions, cyclic compression tests were conducted under sustained loading to representative of residential floor applications. The evolution of deformation behavior and stiffness was monitored over repeated loading cycles.

Dynamic mechanical analysis (DMA) was performed to characterize the viscoelastic behavior of the composite underlayments over temperature and frequency ranges relevant to building applications. Tests were conducted in shear mode. The storage modulus, loss modulus, and loss factor (tan δ) were measured over a temperature range of −20 to 80 °C. Frequency sweep tests (0.1–100 Hz) were additionally performed at room temperature to evaluate the sensitivity of damping behavior to dynamic excitation representative of impact loading.

The measurements were intended to characterize the load-bearing capacity and deformation behavior of the underlayment, which are critical for assessing mechanical compatibility with rigid finishing layers in layered floating floor systems. Cyclic compression tests were conducted to evaluate deformation stability under repeated loading representative of residential service conditions. A sinusoidal compressive stress cycling between 3 kPa (minimum) and 30 kPa (maximum) was applied at 2 Hz (R = 0.1). The peak stress of 30 kPa conservatively represents the combined dead load of a 30 mm gypsum screed with floor covering (~2 kPa), the residential live load per GB 50009 (2.0 kPa), and a dynamic amplification factor for repeated footfall impact. It should be noted that continuous loading imposes higher effective severity than intermittent real-service loading [53]. The stress–strain response was monitored at selected cycle intervals to track changes in deformation behavior and stiffness.

### 2.5. Microstructure Characterization

The microstructure of the composite underlayments was characterized by scanning electron microscopy (SEM). Specimens were cryo-fractured to preserve internal microstructural features and subsequently sputter-coated with a thin conductive layer prior to imaging.

SEM micrographs were employed to evaluate the dispersion of rubber particles and quartz sand within the polymer matrix, the quality of interfacial bonding, and the presence of micro-pores. These microstructural features were examined in relation to the observed mechanical and acoustic performance, with particular attention to their potential role in multi-scale energy dissipation within the composite core.

### 2.6. Acoustic Testing Methods

#### 2.6.1. Laboratory-Scale Sound Insulation Measurements

**Figure 3:**
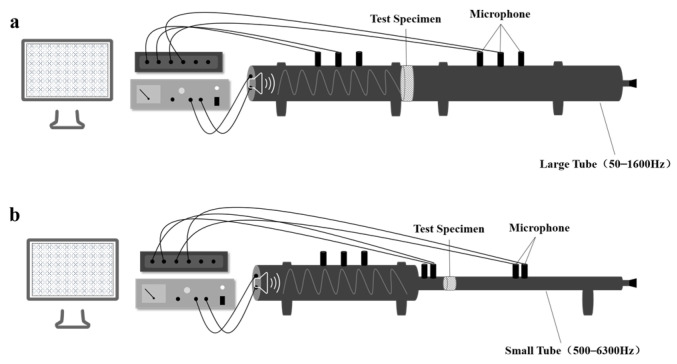
(**a**) Schematic illustration of the laboratory-scale acoustic insulation performance testing setup for low-frequency measurements; (**b**) testing setup for high-frequency measurements.

The normal-incidence sound transmission loss (STL) of the composite underlayment was measured by the two-microphone transfer-function method. The experimental setup comprised an impedance tube (Model AWA8551, Hangzhou Aihua Instruments Co., Ltd., Hangzhou, China), a multi-channel noise analyzer (Model AWA6290M, Hangzhou Aihua Instruments Co., Ltd., Hangzhou, China), and transfer-function analysis software (Model AW6290 Signal Analysis Software, Version 4.0.2, Hangzhou Aihua Instruments Co., Ltd., Hangzhou, China). All measurements were performed at a constant temperature of 23 ± 1 °C. The impedance-tube system incorporated two interchangeable tubes: a 100 mm diameter tube covering the 50–1600 Hz range and a 20 mm diameter tube covering the 500–6300 Hz range. The procedure complied with the national standard GB/T 18696.2-2002 [54] (ISO 10534-2:1998 [55]), Acoustics—Determination of sound absorption coefficient and impedance in impedance tubes—Part 2: Transfer-function method. A schematic diagram of the impedance-tube apparatus is shown in Figure 3. Prior to testing, the microphones were calibrated with an AWA6223 sound level calibrator (Hangzhou Aihua Instruments Co., Ltd., Hangzhou, China). Each sample group was measured in triplicate, and the mean value was adopted as the STL of the corresponding specimen. The overall test frequency range was 50–6300 Hz.

**Figure 4:**
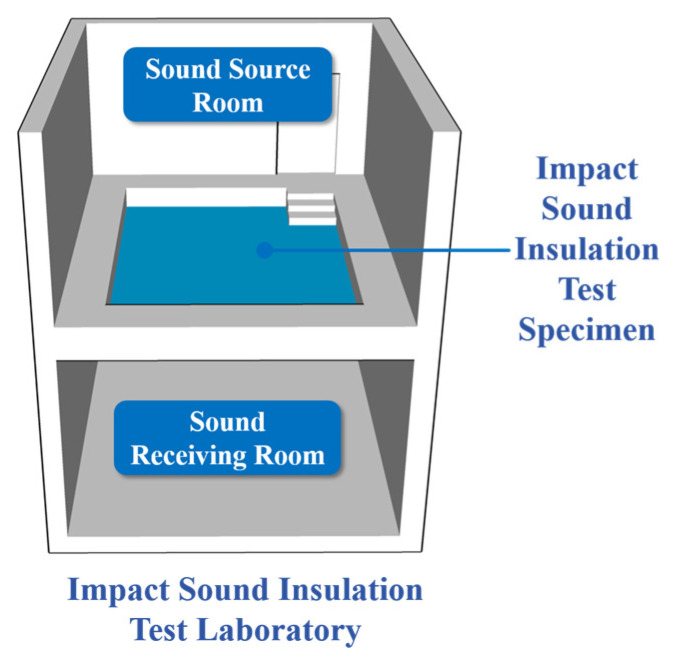
Photograph of the laboratory reverberation room used for impact sound measurements.

The impact sound insulation performance of the floor assembly was evaluated by the standard tapping-machine method under controlled laboratory conditions (temperature 23 ± 2 °C, relative humidity 50 ± 5%). The test instruments included Brüel & Kjær (B&K) sound leveler meter, tapping machine and microphone. The measurement system consisted of a source room (reverberation chamber), a receiving room (semi-anechoic chamber), and the floor specimen under investigation. The test protocols followed GB/T 19889.6-2005 [56] (ISO 140-6:1998 [57]), Acoustics—Measurement of sound insulation in buildings and of building elements—Part 6: Laboratory measurements of impact sound insulation of floors, and GB/T 19889.7-2022 [58] (ISO 16283-2:2020 [59]), Acoustics—Measurement of sound insulation in buildings and of building elements—Part 7: Field measurements of impact sound insulation of floors. It should be noted that GB/T 19889.7-2022 is the Chinese national adoption of ISO 16283-2:2020, and the tapping machine specification (five hammers, 500 g per hammer, 40 mm drop height) prescribed therein is identical to that defined in the ISO 16283-2 and ISO 10140 series [60]. The tapping machine was positioned at the four corners of the test floor (at least 1 m from each wall), and the microphone was placed at a height of 0.5 m above the floor surface, the schematic layout of the impact sound insulation test is illustrated in Figure 4. Before measurement, all microphones were calibrated using an AWA6223 (Hangzhou Aihua Instruments Co., Ltd., Hangzhou, China) sound level calibrator. Impact sound pressure levels were analyzed in 1/3-octave bands over the frequency range of 50–5000 Hz.

#### 2.6.2. Full-Scale Field Measurements

Full-scale field measurements were conducted in an occupied residential building. Impact sound pressure levels were first measured on the bare structural slab (pre-installation baseline). Following the baseline measurement, the composite underlayment was installed according to standard construction procedures, and impact sound pressure levels were re-measured at 1, 7, and 28 days after installation. The pre-installation measurement served as the reference condition against which the post-installation performance was evaluated.

The same Brüel & Kjær (B&K) sound leveler meter, tapping machine and microphone configuration as employed in the laboratory setup was used throughout the field measurements to ensure consistency between laboratory and in situ data. Field measurements were used to evaluate the consistency between laboratory-scale results and in situ performance, as well as to observe mechanical stability of the finished layers during the monitoring period.

The gypsum screed thickness was 30 mm for both installations. Both screeds were cast under identical ambient conditions in the same residential building (same floor, adjacent rooms) and were subjected to identical normal residential occupancy loading, with no artificial or accelerated loading applied. The only variable was the underlayment material. Inspections were conducted at 1, 3, 6 and 12 months post-installation; at each inspection, crack presence, width (crack width gauge, 0.1 mm resolution), and areal density were documented.

### 2.7. Data Analysis and Reproducibility

All tests were conducted on at least 3 replicate specimens for each formulation. Mean values and standard deviations are reported. Measurement uncertainty and repeatability were evaluated in accordance with relevant standards. This approach ensures that the reported results are representative and reproducible for practical building applications.

## 3. Results

### 3.1. Physical Properties of the Composite Underlayments

**Figure 5:**
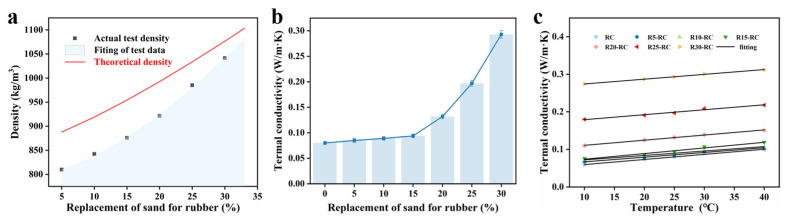
(**a**) theoretical and actual densities of composite underlayments with different sand content; (**b**) the thermal conductivities of different composite underlayments at 25 °C; (**c**) theoretical and actual thermal conductivities of different composite underlayments at different temperature.

The bulk density and thermal conductivity of the composite underlayments with varying quartz sand substitution ratios are summarized in Figure 5. As the quartz sand content increased, the bulk density of the composites exhibited a systematic increase, ranging from 815 to 1042 kg/m^3^ (Figure 5a). The measured densities closely matched the theoretical values calculated using the linear mixing rule, indicating uniform dispersion of the constituent phases. Thermal conductivity results are presented in Figure 5b,c. The measured thermal conductivity values remained within the range acceptable for interior floor applications and showed a moderate increase with increasing sand content. No abrupt changes in thermal behavior were observed across the investigated compositions, suggesting that the incorporation of rigid mineral fillers did not compromise the thermal suitability of the composite underlayments for building applications.

**Figure 6:**
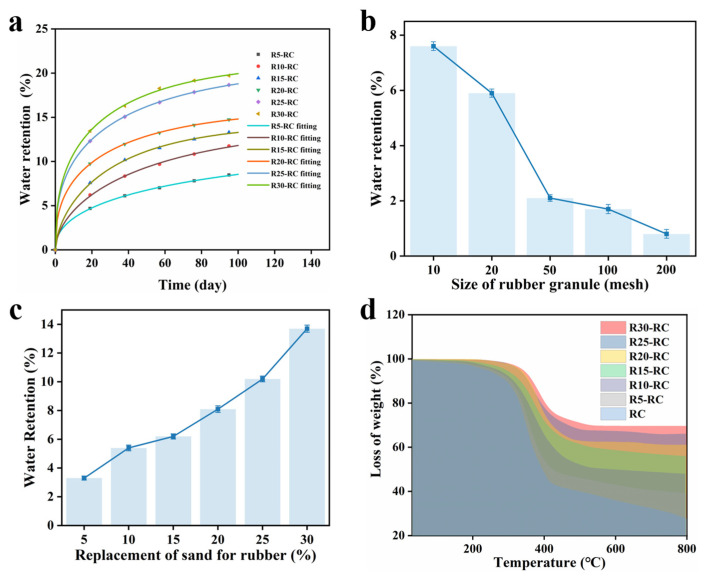
(**a**) Effect of rubber particle size on water retention; (**b**) effect of quartz sand substitution ratio on water retention; (**c**) water absorption equilibrium behavior of different composite underlayments; (**d**) the TGA curve of different composite underlayments.

Water retention behavior is illustrated in Figure 6. As shown in Figure 6b, increasing rubber particle size resulted in a gradual decrease in water retention, which can be attributed to the reduced specific surface area and lower water affinity of coarser rubber particles. In contrast, Figure 6c indicates that increasing the quartz sand substitution ratio led to a moderate increase in water retention, likely associated with the formation of additional micro-pores and capillary pathways introduced by the mineral filler phase.

**Figure 7:**
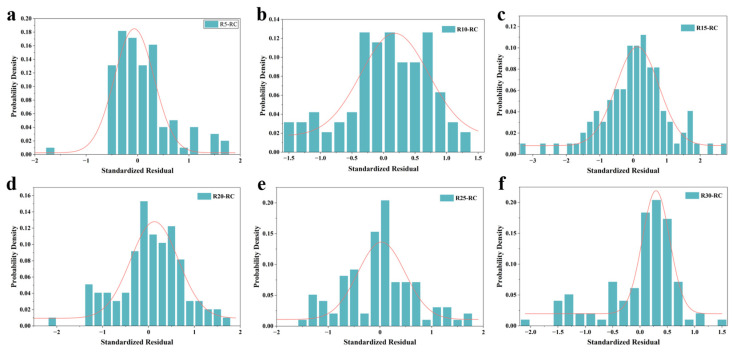
(**a**) R5-RC; (**b**) R10-RC; (**c**) R15-RC; (**d**) R20-RC; (**e**) R25-RC; (**f**) R30-RC.

The time-dependent water absorption behavior of the composite underlayments is shown in Figure 6a. All samples exhibited a rapid initial water uptake followed by a gradual approach to saturation, indicating diffusion-controlled absorption behavior. The experimental data were fitted using a three-parameter model, WR(t) = a (1 − exp(−bt))^c, yielding coefficients of determination (R^2^) exceeding 0.99 for all formulations (Table 2). Furthermore, the standardized residuals were normally distributed (Figure 7), confirming the reliability of the model. Importantly, the equilibrium water retention values remained within a range compatible with interior floor applications, indicating no excessive moisture sensitivity under the tested conditions.

The physical significance of the three parameters in the fitted model is discussed as follows. Parameter a represents the equilibrium (saturation) water retention capacity. Physically, “a” scales with the total accessible pore volume and the overall hydrophilicity of the composite constituents. The systematic increase in “a” with increasing quartz sand content (Table 2, from 11.94% for R5-RC to 21.34% for R30-RC) is consistent with the introduction of additional micro-pores and hydrophilic capillary pathways by the mineral filler phase, as also reflected in the water absorption curves (Figure 6c). Parameter “b” governs the initial rate of water uptake; a larger “b” corresponds to more rapid absorption kinetics and is primarily influenced by surface wettability and the connectivity of the pore network during the early stages of water ingress. Parameter “c” controls the shape of the absorption curve, specifically the transition behavior between initial rapid uptake and the gradual approach to saturation equilibrium. The fitted values of “c” (0.36–0.59) are consistently below unity, indicating diffusion-controlled (Fickian-like) water absorption behavior in which the absorption rate decreases progressively as the saturation state is approached. This is characteristic of porous composites in which water transport is dominated by capillary diffusion rather than polymer relaxation or swelling.

Thermal stability was further evaluated by thermogravimetric analysis (TGA). Representative TGA curves are shown in Figure 6d, and characteristic thermal stability parameters are summarized in Table 3. With increasing quartz sand content, the temperature corresponding to 5% mass loss (T_5_%) increased systematically, while the overall mass loss decreased. These trends indicate improved thermal stability of the composite underlayments due to the increasing proportion of thermally stable mineral filler.

### 3.2. Mechanical Behavior and Deformation Characteristics

**Figure 8:**
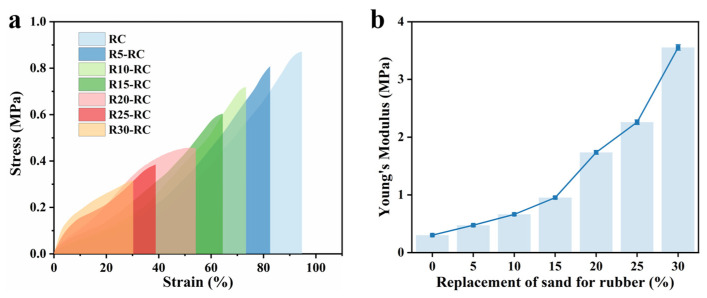
(**a**) compressive stress–strain curves of different composite underlayments; (**b**) Young’s modulus of different composite underlayments.

Representative compressive stress–strain curves of the composite underlayments are shown in Figure 8a. All formulations exhibited an initial linear elastic region followed by a gradual transition to nonlinear deformation at higher strain levels. As the quartz sand content increased, the overall stiffness of the composites increased accordingly.

The apparent elastic modulus extracted from the linear region of the stress–strain curves is summarized in Figure 8b. A monotonic increase in elastic modulus was observed with increasing sand substitution ratio, demonstrating that the stiffness of the composite underlayments can be effectively regulated through compositional control.

**Figure 9:**
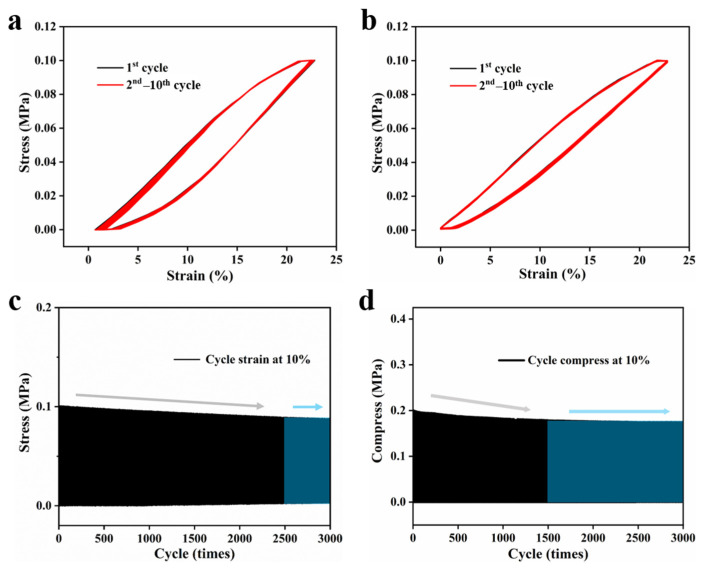
(**a**) 10 strain cycles of R20-RC for 10 min interval; (**b**) 10 strain cycles of R20-RC for 30 min interval; (**c**) 3000 strain cycles of R20-RC; (**d**) 3000 compress cycles of R20-RC.

The deformation stability of the composite underlayments under repeated loading is illustrated in Figure 9. Cyclic compression tests conducted at different time intervals showed stable stress–strain responses, with no abrupt changes in stiffness observed over the test duration. Even after 3000 loading cycles, the composites maintained consistent deformation behavior.

Compared with conventional XLPE foam underlayments tested under identical conditions, the composite materials exhibited reduced permanent deformation under sustained and cyclic loading. This behavior indicates improved mechanical stability and load-bearing capacity, which are critical for maintaining compatibility with rigid finished layers in floating floor systems.

**Figure 10:**
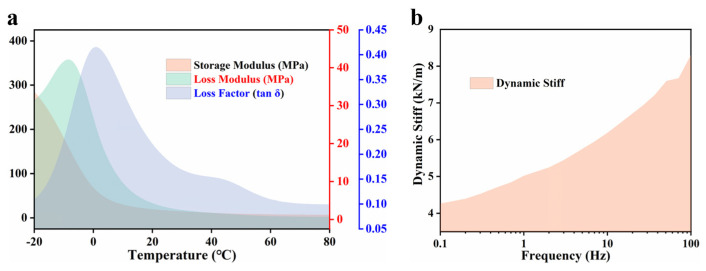
(**a**) the DMA results of R20−RC with different temperature; (**b**) the dynamic stiff of R20−RC with different frequency.

Dynamic mechanical analysis results are presented in Figure 10. The composite storage modulus gradually declines from 280 MPa at −20 °C to 10 MPa at 80 °C, representing a typical transition from a low-temperature glassy rigid state to a high-temperature flexible rubbery state. Within conventional indoor service temperatures, the material retains moderate and stable stiffness, offering adequate load-bearing capability and long-term deformation resistance while maintaining good compatibility with rigid gypsum screeds. This mechanical characteristic well meets the stiffness requirement of the viscoelastic interlayer in CLD-inspired systems.

The loss modulus results reflect temperature-dependent interfacial shear and internal friction, providing direct mechanical evidence that low-frequency vibration energy is primarily dissipated via shear deformation—the fundamental principle underlying the CLD-inspired mechanism.

The loss factor decreases from 0.41 to 0.1 across the tested temperature range, yet remains steadily above 0.2 under typical residential indoor conditions. This stable damping behavior verifies that dispersed rubber particles function as effective localized viscoelastic damping domains, enabling efficient low-frequency noise attenuation and further supporting the rationality of the proposed CLD-inspired design.

### 3.3. Microstructural Characteristics

**Figure 11:**
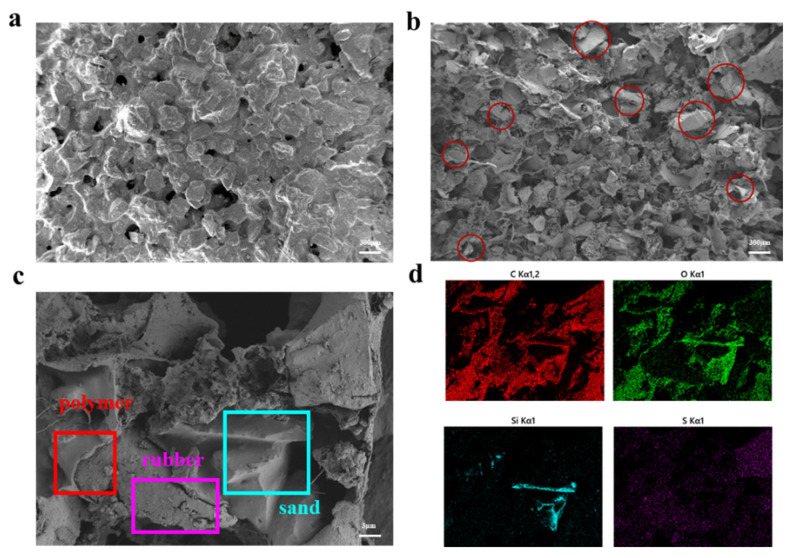
(**a**) the microstructural morphology of R20-RC; (**b**) the cross-section of R20-RC; (**c**) analysis of different interfaces in R20-RC microstructure; (**d**) analysis of element distribution in R20-RC microstructure.

Representative SEM images of the composite underlayments are shown in Figure 11. The micrographs reveal a heterogeneous microstructure consisting of rubber particles embedded within a continuous polymer matrix, interspersed with angular quartz sand particles. The constituent phases are well distributed throughout the composite, and no large-scale agglomeration or phase separation is observed.

At the rubber–matrix and sand–matrix interfaces, continuous interfacial contact is evident, suggesting adequate bonding between the viscoelastic phase, rigid fillers, and polymer binder. No pronounced interfacial debonding or crack initiation sites are observed within the resolution of the SEM analysis.

In addition, interconnected micro-pores are present within the composite structure. Although the pore size and distribution vary locally, the pores are generally well dispersed throughout the material. This heterogeneous microstructure is consistently observed across multiple specimens, indicating good fabrication reproducibility. The coexistence of viscoelastic rubber particles, rigid mineral fillers, and micro-porous regions provides a microstructural basis for multi-scale deformation and energy dissipation under dynamic loading.

SEM microstructure characterization directly reveals the formation of microscale constrained damping units inside the composite. Recycled rubber particles are uniformly dispersed as independent localized viscoelastic damping domains, while rigid quartz sand and polymer matrix form the surrounding constraint phase. Under impact vibration excitation, relative micro-displacement and interfacial shear deformation occur between the flexible rubber domains and rigid constraint phases, producing hysteretic energy dissipation at the microscopic scale. This microscopically distributed shear-dominated dissipation behavior is consistent with the core energy dissipation principle of classical CLD. Energy-dispersive X-ray spectroscopy (EDS) elemental mapping of the R20-RC composite is presented in Figure 11d. The elemental distribution reveals distinct spatial patterns: silicon (Si) is predominantly localized in angular regions corresponding to quartz sand particles, carbon (C) is uniformly distributed throughout the polymer–rubber matrix, and oxygen (O) is associated with both the quartz sand (SiO_2_) and the polyester binder. Notably, the Si-rich domains are well dispersed within the continuous C-rich matrix, with no evidence of large-scale agglomeration or elemental segregation, confirming the homogeneity of the composite at the microscale. This uniform dispersion of rigid quartz sand particles within the viscoelastic matrix is of particular significance for the proposed CLD-inspired energy dissipation mechanism: each quartz sand particle, intimately embedded in the surrounding polymer–rubber phase, is positioned to provide localized mechanical constraint under impact-induced deformation, thereby promoting shear deformation and energy dissipation within adjacent viscoelastic rubber domains.

### 3.4. Impact Sound Insulation Performance Measurement Results

**Figure 12:**
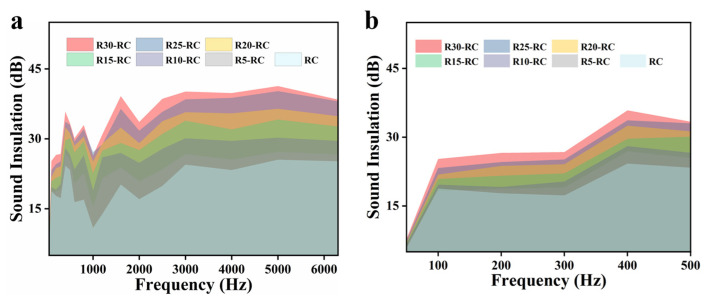
The acoustic insulation performance of different composite underlayments; (**a**) 50–6300Hz; (**b**) 50–500Hz.

**Figure 13:**
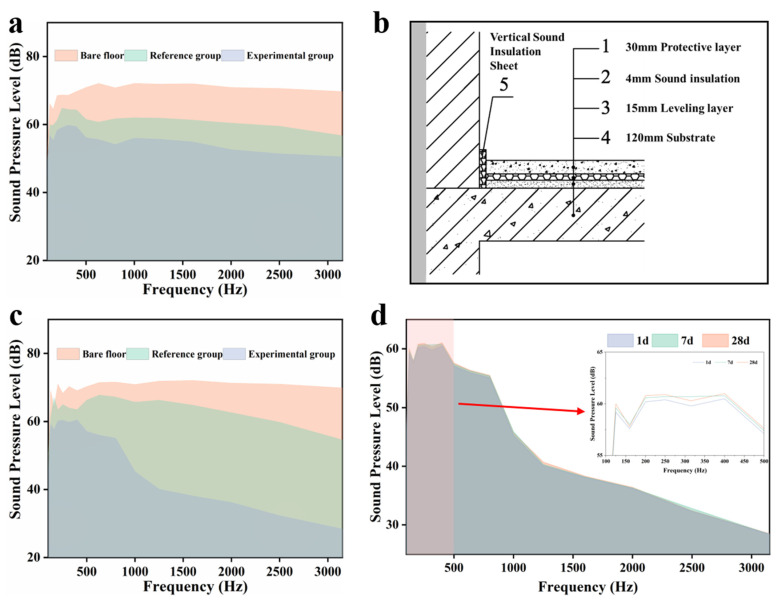
(**a**) the test results of the model laboratory; (**b**) floating floor slab construction diagram; (**c**) the test results of the residential house; (**d**) comparison of 1 d, 7 d and 28 d test results of composite sound insulation cushion, with the low-frequency range (50–500 Hz) enlarged in the inset.

The laboratory-scale impact sound insulation performance of floating floor assemblies incorporating the composite underlayments is presented in Figure 12 and Figure 13. Compared with the bare concrete slab, all composite-based floating floor configurations resulted in a clear reduction in normalized impact sound pressure levels across the measured frequency range.

As the quartz sand substitution ratio increased, the impact sound insulation performance measured using the impedance tube exhibited a corresponding improvement. This trend is particularly evident in the low-frequency range below 250 Hz, as shown in Figure 12b, where conventional soft underlayments typically show limited effectiveness.

Under identical construction conditions, floating floor assemblies incorporating the composite underlayments consistently exhibited lower normalized impact sound pressure levels than those using conventional XLPE foam underlayments. The magnitude of improvement varied with material composition, reflecting differences in mass density, stiffness, and damping behavior among the tested formulations.

These laboratory-scale results indicate that the proposed composite underlayments provide enhanced low-frequency impact sound insulation relative to conventional soft underlay materials under controlled conditions.

The results of full-scale field measurements conducted in an occupied residential building are shown in Figure 13. Installation of the composite underlayment resulted in a measurable reduction in impact sound pressure levels compared with the pre-installation condition. Figure 13c presents the measured impact sound pressure levels before installation (bare slab) and after installation (composite underlayment at 28 days). Compared with the pre-installation baseline, the installation of the composite underlayment resulted in a measurable reduction in impact sound pressure levels across the measured frequency range, with effective improvement observed in the low-frequency range below 250 Hz. Specifically, the normalized impact sound pressure levels for the bare concrete slab, the control group, and the experimental group (composite underlayment) were 73 dB, 65 dB, and 58 dB, respectively, corresponding to a 10.77% reduction relative to the control group and a 20.55% reduction relative to the bare slab. The trends observed in the field measurements are consistent with the laboratory-scale results, particularly with respect to improved performance in the low-frequency range. Although absolute sound pressure levels differ between laboratory and in situ measurements due to boundary conditions and structural variability, the relative performance trends remain comparable. As shown in Figure 13d, the sound insulation performance of the composite-underlayment floating floor remained stable across the 1-, 7-, and 28-day measurements, with negligible variation, indicating robust acoustic stability over time.

**Figure 14:**
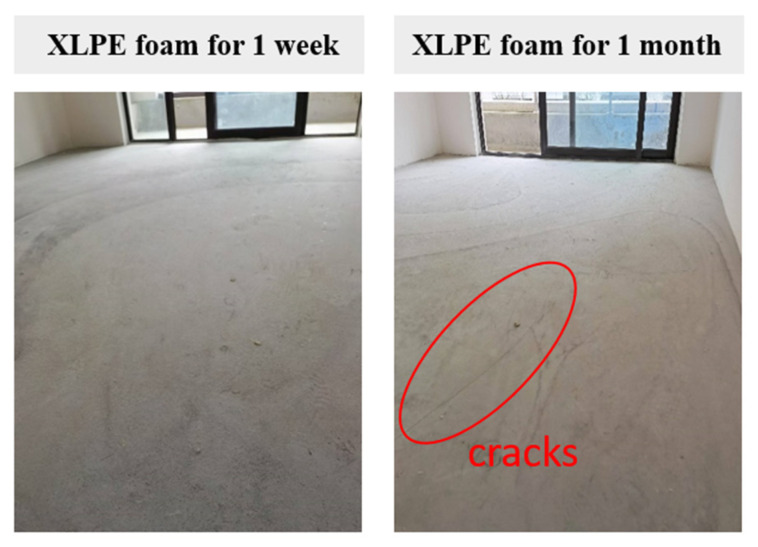
Surface condition of gypsum screeds installed over XLPE foam underlayments under long-term loading.

**Figure 15:**
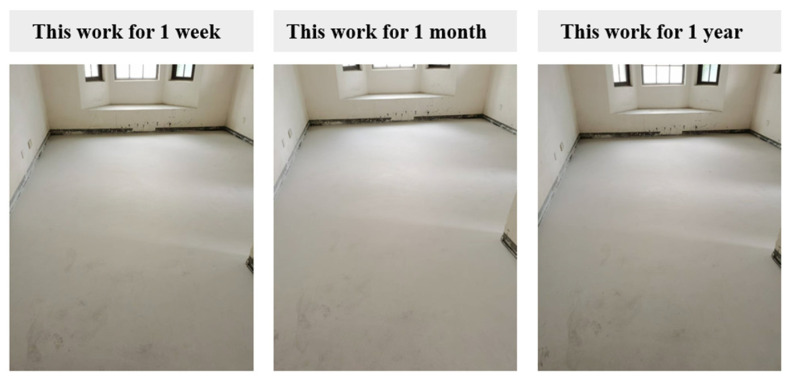
Surface condition of gypsum screeds installed over the composite underlayments under long-term loading.

Over the 12-month monitoring period, the XLPE underlayment screed developed visible cracks (first observed at 1 month; max width 1.8 mm, density 5–10 cracks/m^2^ at endpoint), while the R20-RC composite underlayment screed remained free of cracking, delamination, or surface damage throughout (Figure 14 and Figure 15). These observations indicate substantially improved structural compatibility under real service conditions.

## 4. Discussion

### 4.1. Interpretation of Impact Sound Reduction from a CLD-Inspired Perspective

To avoid over-interpretation of the CLD concept, this paper makes an explicit distinction between the developed composite underlayment and traditional macroscopic constrained layer damping structures. Mechanistically, both designs dissipate low-frequency vibration energy relying on shear deformation of viscoelastic components and the constraint effect of rigid phases. Nevertheless, classical CLD adopts a standard macroscopic three-layer laminated structure, while our developed composite is a multiphase heterogeneous material with microscopically distributed constrained damping domains, rather than following a conventional macroscopic layered CLD layout.

The experimental results demonstrate that the composite underlayments provide enhanced low-frequency impact sound insulation compared with conventional soft underlay materials. This behavior can be interpreted from a constrained-layer-damping-inspired (CLD-inspired) perspective, in which energy dissipation arises from the combined action of viscoelastic deformation, interfacial shear, and constrained motion of compliant phases between relatively rigid layers [38].

**Figure 16:**
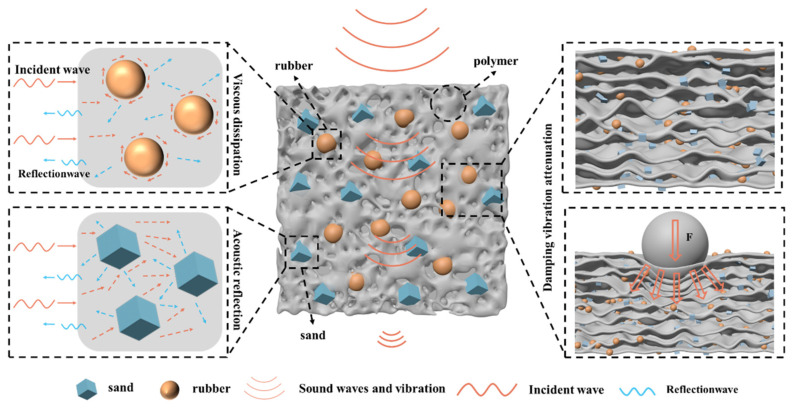
Mechanism diagram of propagation and dissipation of sound waves inside the composite underlayment. The arrow indicates the force transmission path, and ‘F’ denotes the applied load.

Unlike classical CLD configurations consisting of discrete layered assemblies, the present composite underlayments exhibit a spatially distributed CLD-like mechanism at the material scale. Rubber particles embedded within the polymer matrix act as localized viscoelastic domains, while quartz sand particles and the rigid finishing layers provide partial mechanical constraint. Under impact excitation, differential deformation between these phases induces interfacial shear and hysteretic energy dissipation, which is particularly effective at low frequencies where purely elastic soft materials tend to be less efficient. As shown in Figure 16, the acoustic insulation principle of floating floor systems depends on the reflection and transmission of sound waves at multiple interfaces between different media. When sound waves pass through the elastic interlayer, multiple reflections occur within the viscoelastic medium, dissipating a significant amount of energy and thereby enhancing acoustic insulation performance.

The observed improvement in low-frequency impact sound insulation is therefore attributed to the synergistic combination of increased mass density, regulated stiffness, and stable damping behavior, rather than to a single dominant factor. This interpretation is consistent with the dynamic mechanical analysis results and the microstructural observations presented in Section 3.

The elemental distribution map (Figure 11d) provides direct microstructural evidence supporting this interpretation: the uniform dispersion of Si-rich quartz sand domains within the C-rich polymer–rubber matrix ensures that rigid inclusions are available throughout the composite to provide localized mechanical constraint, while the absence of phase segregation confirms that the viscoelastic and rigid phases are intimately coupled at the microscale.

The key similarities and differences between classical CLD configurations and the proposed composite are summarized in Table 4.

### 4.2. Role of Material Composition and Stiffness Regulation

The results indicate that material composition plays a critical role in balancing acoustic performance and mechanical stability. Increasing the quartz sand substitution ratio leads to higher stiffness and mass density, which contributes to improved impact sound insulation performance. However, excessive stiffening may reduce the compliance required for effective vibration isolation, highlighting the need for compositional optimization.

The monotonic increase in elastic modulus observed in Section 3.2 demonstrates that the stiffness of the composite underlayments can be systematically regulated through compositional design. Within the investigated range, the selected formulations achieve a favorable balance between load-bearing capacity and damping effectiveness, enabling both acoustic improvement and structural compatibility with rigid finished layers.

Compared with conventional XLPE foam underlayments, the composite materials exhibit reduced permanent deformation and improved deformation stability under sustained and cyclic loading. This mechanical robustness is particularly important for floating floor systems, where long-term load-induced damage and screed cracking remain key practical concerns.

Although a side-by-side DMA comparison with XLPE foam under identical experimental conditions was not feasible within the scope of this study, the available literature provides a useful reference for contextualizing the damping performance of the composite underlayment. Published DMA studies on closed-cell polyolefin foams have consistently reported loss factors (tan δ) in the range of approximately 0.10–0.15 at ambient temperatures (20–30 °C) [61]. These values are substantially lower than the tan δ > 0.2 measured for the R20-RC composite under comparable temperature conditions (Figure 10a). While differences in test configuration (e.g., tensile vs. shear mode, frequency, and specimen geometry) preclude a strictly quantitative comparison, the order-of-magnitude difference is consistent with the fundamentally distinct energy dissipation mechanisms in the two material classes: viscoelastic shear and interfacial friction in the rubber-based composite versus modest intrinsic damping in the low-density polyolefin foam.

### 4.3. Structural Compatibility and Deformation Stability

Beyond acoustic performance, the mechanical behavior of the underlayment plays a critical role in the long-term reliability of floating floor systems. The reduced permanent deformation observed in the composite underlayments under sustained and cyclic loading suggests improved deformation stability compared with soft foam materials. This behavior is particularly relevant for brittle finished layers, such as gypsum screeds and ceramic tiles, which are sensitive to differential settlement and stress concentrations [62,63].

Field observations conducted over the monitoring period indicate that the composite underlayments provide sufficient load-bearing support to prevent surface cracking under typical residential service conditions. These findings support the view that underlayment design should be evaluated not only in terms of initial acoustic performance, but also with respect to mechanical compatibility and long-term deformation behavior.

### 4.4. Consistency Between Laboratory and Field Performance

The agreement between laboratory-scale measurements and full-scale field observations provides further support for the practical applicability of the proposed composite underlayments. Although absolute impact sound pressure levels differ due to boundary conditions, construction tolerances, and structural variability, the relative performance trends remain consistent.

Importantly, the field measurements confirm that the improved low-frequency impact sound insulation observed under controlled laboratory conditions can be translated to real building environments. The absence of visible cracking or surface damage in the finished layers further indicates improved structural compatibility under long-term service conditions.

These findings highlight the importance of evaluating floating floor underlayments not only in terms of acoustic performance but also with respect to mechanical durability and system-level compatibility.

### 4.5. Limitations and Outlook

Despite the promising results, several limitations should be acknowledged. First, the present study focuses on a limited range of material compositions and thicknesses. Further optimization may be achieved by expanding the design space to include alternative filler types, binder formulations, and multilayer configurations.

Second, the present study adopts a CLD-inspired conceptual framework to interpret the observed acoustic performance but does not provide a direct quantitative comparison of shear strain energy between the proposed composite and a classical CLD configuration. Such a comparison would require finite element modeling incorporating full viscoelastic material constitutive parameters or direct strain field measurements (e.g., via digital image correlation), both of which represent directions for future investigation. The CLD-inspired interpretation advanced herein should therefore be regarded as a physically motivated conceptual framework rather than a rigorous mechanistic validation.

Finally, long-term environmental effects such as temperature cycling, moisture exposure, and aging were not systematically investigated and should be addressed in future work to fully assess durability under diverse service conditions.

## 5. Conclusions

This study proposes and evaluates a composite floating floor underlayment composed of recycled rubber particles, polymer resin, and quartz sand, targeting improved low-frequency impact sound insulation in conjunction with long-term mechanical stability. Based on the experimental results and field observations, the following conclusions can be drawn:

1. The composite underlayments exhibit controllable physical and mechanical properties through compositional design. Increasing the quartz sand substitution ratio leads to higher stiffness and mass density, while stable deformation behavior is maintained under sustained and cyclic loading conditions;
2. Compared with conventional XLPE foam underlayments, the proposed composite materials provide enhanced low-frequency impact sound insulation, particularly in the frequency range below 250 Hz, where conventional soft underlays typically show limited effectiveness;
3. Microstructural observations and dynamic mechanical analysis support a CLD-inspired interpretation of the impact sound reduction mechanism, in which distributed viscoelastic domains and mechanically constrained phases contribute to effective energy dissipation under impact excitation;
4. Full-scale field measurements and long-term service observations confirm that the composite underlayments achieve satisfactory structural compatibility with rigid finished layers, with no visible cracking or surface damage observed during the monitored service period.

Overall, the results demonstrate that integrating CLD-inspired energy dissipation mechanisms into floating floor underlayment design provides a promising and practical approach for addressing low-frequency impact noise in buildings while ensuring mechanical durability.

## Figures and Tables

**Table 1:** Thermal conductivity of composite underlayments with different rubber particle sizes.

Size	10 Mesh	20 Mesh	50 Mesh	100 Mesh	200 Mesh
Thermal Conductivity W/(m⋅K)	0.315	0.247	0.132	0.106	0.083

**Table 2:** Fitted parameters for the water absorption equilibrium model.

Sample	Equation Parameters			
	a	b	c	R^2^
R5-RC	11.94	0.00607	0.4253	0.9961
R10-RC	11.53	0.02031	0.3988	0.9990
R15-RC	14.31	0.02137	0.5893	0.9980
R20-RC	16.09	0.01633	0.3816	0.9995
R25-RC	20.94	0.01338	0.3558	0.9995
R30-RC	21.34	0.01795	0.3734	0.9963

**Table 3:** Thermal stability parameters obtained from TGA.

Sample	RC	R5-RC	R10-RC	R15-RC	R20-RC	R25-RC	R30-RC
T_5%_	214.9	256.7	271.3	293.8	326.9	331.3	340.8
Overall change (%)	72.4	60.8	52.1	44.1	38.9	33.9	30.4

**Table 4:** Comparison of key features between classical CLD systems and the proposed composite underlayment.

Feature	Classical CLD System	Proposed Composite	Remarks
Constraining architecture	Continuous high-stiffness lamina (e.g., metal sheet)	Dispersed quartz sand particles + rigid finishing screed	Architecturally distinct; functional analogy from localized constraint of viscoelastic domains
Viscoelastic medium	Continuous viscoelastic film	Dispersed rubber domains in polymer matrix	Multi-scale vs. bulk deformation
Primary deformation mode	Macroscopic shear of viscoelastic core	Localized shear of rubber domains + interfacial friction at particle–matrix boundaries	Additional dissipation pathways in the composite
Energy dissipation mechanism	Bulk viscoelastic shear loss	Combined viscoelastic loss, interfacial friction, and multi-scale stress redistribution	More complex, multi-mechanism dissipation
Key design parameters	Layer thickness, Young’s moduli, loss factor	Filler content, particle size distribution, matrix properties	Compositional rather than geometric tuning
Fabrication	Lamination or co-curing of distinct layers	Particulate mixing and casting	Simpler, single-step fabrication

## Data Availability

The original contributions presented in this study are included in the article. Further inquiries can be directed to the corresponding author.

## Abbreviations

The following abbreviations are used in this manuscript:

CLD	Constrained-Layer-Damping
XLPE	Cross-linked Polyethylene
DMA	Dynamic Mechanical Analysis
SEM	Scanning Electron Microscopy
TGA	Thermogravimetric Analysis
